# Adolescents' Perspectives on Self‐Regulation and Need for Change in Digital Media Use: A Focus Group Study

**DOI:** 10.1002/jad.70220

**Published:** 2026-07-06

**Authors:** Luka Todorovic, Annabel Bogaerts, Helle Larsen

**Affiliations:** ^1^ Developmental Psychology University of Amsterdam Amsterdam North Holland the Netherlands

**Keywords:** adolescents, digital media use, problematic social media use, psychological needs, qualitative research, self‐regulation

## Abstract

**Introduction:**

Research on digital media use and adolescents' development and well‐being remains inconclusive, and adolescents' own perspectives on these issues are largely unexplored. Nevertheless, concrete policy measures are being implemented to regulate adolescents' digital media use. Therefore, this study examined adolescents' own perspectives on the effects of digital media use, the cognitive processes relating to their self‐regulation of digital media use, and their perceived need for change in how they use digital media.

**Methods:**

We conducted 10 focus group interviews with Dutch early‐to‐middle adolescents (*N* = 78; 56% girls; *M*_age_ = 14.1, *SD*_age_ = 1.1) from two secondary schools in the Netherlands. Data were analysed using codebook thematic analysis with a combination of inductive and deductive coding informed by the triple‐pathway model.

**Results:**

Adolescents expressed their appreciation for various benefits that digital media enable them, but also concerns about many challenges that they face online. Regarding mechanisms, they discussed how digital media can capture attention, distort time perceptions, and create challenges with self‐regulation. When discussing the need to change their digital media use, adolescents emphasised that not only individual efforts in self‐regulation, but also shared (with parents and peers) and structural (e.g., school‐level) solutions may help to prevent exclusion and other risks.

**Conclusion:**

Adolescents desire feasible ways to balance digital media use that preserve agency and relatedness while reducing negative effects. The findings have implications for research, education, parenting, and policy focused on supporting adolescents' development in digital contexts.

There are ongoing debates about the impact of digital media, such as social media and video games, on adolescents' development and well‐being (Montag et al. 2024; Pearson 2025). However, it is also acknowledged that the research on digital media use in adolescents is not keeping pace with technological developments to provide sufficient evidence for informing sound policy on digital media regulation (Orben and Matias 2025): concrete policy decisions are being implemented, such as smartphone bans in schools and social media age restrictions, and given the challenges associated with obtaining conclusive quantitative evidence, systematic consideration of adolescents' own perspectives is needed to represent those directly affected and to access contextual insight that quantitative approaches have not been able to provide. Findings indicate that a variety of positive and negative effects on adolescents occur in relation to social media use (Valkenburg et al. 2021; Nagata et al. 2025), but also in relation to other digital media behaviours such as gaming or pornography use (Alanko 2023; Paulus et al. 2024).

Evidence for negative consequences on well‐being is most robust for dysregulated patterns of use, such as problematic social media use (Huang 2020; Shannon et al. 2022) and problematic gaming (van der Neut et al. 2023). Furthermore, the effects of digital media use on well‐being depend on individual factors (Valkenburg and Peter 2013), such as sex and cognitive profile. Therefore, it is difficult to draw general conclusions regarding the appropriate and beneficial forms of digital media use for adolescents. The present study investigated adolescents' own perspectives on digital media use, their experiences of cognitive processes potentially underlying negative effects of digital media use, and their views on feasible and desirable change in how they use digital media. Digital media use was conceptualised as common everyday use of screen‐based devices, with social media and gaming as main examples of digital media engagement for this specific age group.

The variety of perceived effects of digital media use has already been examined in previous qualitative research with adolescents (e.g., Popat and Tarrant 2022), suggesting that adolescents mainly use digital media for purposes surrounding relational interactions, self‐expression, exploration, and browsing, and that within each of these domains, positive and negative effects occur (Weinstein 2018; West et al. 2021, 2023). Focus groups show that Dutch adolescents experience heterogeneous and dual affective responses to social media, which can be positive, negative, and mixed (van der Wal et al. 2024), and that UK adolescents exhibit a variety of motives for social media use, where control motives may underlie problematic social media use (Throuvala et al. 2018). Differences in digital media effects also emerge by sex in U.S. and U.K. samples (Twenge and Martin 2020). Literature suggests that girls appear more vulnerable to pressures related to self‐presentation and social standing (Belfort et al. 2025). Boys who use social media intensively are found to be more susceptible to impulsive decision‐making (Hayashi and Glodowski 2021), consistent with their higher levels of impulsive behaviours during adolescence (Carvalho et al. 2023; Pante et al. 2024) and longitudinal evidence showing hyperactivity/impulsivity to precede problematic social media use among boys (Todorovic et al. 2025).

While the variety of positive and negative effects of digital media use on adolescents has been studied relatively extensively with both quantitative and qualitative designs (Haddock et al. 2022; Orben 2020), the underlying mechanisms of such effects remain to be understood (Orben et al. 2024). The existing qualitative research has addressed effects, affective responses, and motivational processes, but not how adolescents themselves experience cognitive mechanisms underlying their difficulties with self‐regulation or their perspectives on change in digital media use. Adequate self‐regulation capacities may therefore be one of the key determinants of whether digital media use remains balanced or becomes problematic (Reinecke et al. 2021).

Digital media use may become problematic due to lower self‐regulation capacities (Howard et al. 2024), especially during adolescence (Hrbackova et al. 2025), a critical period for the development of effective self‐regulation (Silvers 2021; Wesarg‐Menzel et al. 2023). In general, adolescents with more symptoms of attention‐deficit/hyperactivity disorder (ADHD), characterised by, among other features, problems of self‐regulation, are found to exhibit more problematic digital media use over time: research points to both unidirectional effects of digital media use on increased ADHD symptoms among adolescents (Boer et al. 2019; Wallace et al. 2023), as well as bidirectional effects between ADHD symptoms and problematic digital media use (Thorell et al. 2022). Such findings suggest that the behavioural dysregulation profile indicated by inattention, impulsivity, and hyperactivity provides a useful framework for exploring dysregulated digital media use patterns among adolescents.

The triple‐pathway model highlights inhibition deficits, reward sensitivity, and temporal processing deficits as the key cognitive factors underlying the behavioural dysregulation profile observed in ADHD (Sonuga‐Barke et al. 2010) and represents one of several possible theoretical lenses for understanding problematic digital media use. We adopt it as a theoretical lens because even subclinical levels of ADHD symptoms are associated with impaired well‐being and problematic digital media use among adolescents (Gostoli et al. 2024; Krauss and Schellenberg 2022) and adults (Aydin et al. 2023), implicating the relevance of the cognitive domains from the triple‐pathway model beyond clinical contexts. Quantitative research has found positive associations between different forms of problematic digital media use and inhibitory deficits (Moretta and Buodo 2021; Wegmann et al. 2020), higher reward sensitivity (Pirrone et al. 2023; Vargas et al. 2019), and temporal processing deficits (Marciano and Camerini 2022). Such findings highlight the need to complement quantitative findings with qualitative research, as existing studies do not yet address how adolescents themselves experience and make sense of such processes in relation to digital media use. Qualitative work is crucial not only for capturing how these cognitive processes may manifest in adolescents' everyday experiences, but also for understanding meanings, motivations, and contextual circumstances that shape how and when digital media use becomes difficult to regulate or a source of negative impact. When digital media use becomes difficult to regulate, such mechanisms may become particularly salient and coincide with adolescents' desire to change their digital media use.

In line with the bioecological model of human development (Bronfenbrenner and Morris 2006), changes in adolescents' digital media use may occur as a result of both individual self‐regulatory capacities and influences across contexts such as family (e.g., parental rules), school (e.g., peer norms), technological development (e.g., platform design), and policy regulation (e.g., age limits on social media use). Some countries have already implemented restrictions on adolescents' access to social media despite the lack of a strong evidence base for the effectiveness of such measures (Blake et al. 2024; McAlister et al. 2024), and the evidence on the effects of smartphone bans which are taking place in schools is also inconclusive (Campbell et al. 2024; Vanluydt et al. 2026). On the other hand, adolescents themselves also adjust their ways of using digital media depending on their needs, individual characteristics, and platform features (Maheux et al. 2024). However, some features of digital media might challenge adolescents' abilities to self‐regulate their digital media use, such as high personalisation, frequent exposure to novel and exciting content, and ease of continual engagement and access (Roberts and David 2025). This means that design adjustments in digital media affordances may be necessary (Sala et al. 2024) in addition to practical support for enhancing self‐regulation of digital behaviour among adolescents (McAlister et al. 2024).

However, without knowing what adolescents themselves think about changing their digital media use and where they see realistic targets for change, efforts to support healthier digital media use may risk misaligning with their actual experiences, values, and developmental needs. This knowledge could provide relevant insights not only into adolescents' vulnerabilities, but also into where they consider change as appropriate, so that the benefits they obtain from digital media use are not taken away and their autonomy is safeguarded, which is a central need in this developmental period (Soenens et al. 2017) and closely intertwined with digital media use (West et al. 2023). Given that mechanisms underlying problematic digital media use may involve distinctive subjective experiences, such as perceived loss of control, preoccupation, or increased craving (e.g., Montag et al. 2024), it is crucial to understand how adolescents themselves make sense of these processes and what they believe is possible or realistic to change. Therefore, adolescents' perspectives on digital media use may deepen our understanding of how digital media affects them and what relevant meanings and contexts they associate with digital media use. This kind of knowledge is necessary for approaching adolescents' digital media use effectively at home, school, and policy levels.

## 1 Current Study

The current study investigated adolescents' perspectives on the effects of digital media use, the underlying cognitive mechanisms that may explain negative effects (i.e., self‐regulation difficulties), and their need for change related to digital media use, with the aim to contribute adolescent‐informed evidence to a field of research and policy that has not fully utilised it. Focus groups of early‐to‐middle adolescents were held in two Dutch secondary schools, with separate groups of girls and boys because homogenous groups can facilitate open discussion of potentially sensitive topics (Greenwood et al. 2014), which is relevant given the observed sex differences in adolescents' digital media use (Twenge and Martin 2020). We expected findings to illustrate how adolescents themselves experience the cognitive processes underlying self‐regulation difficulties and what they consider as feasible and desirable change – providing qualitative evidence on dimensions that existing research has not yet addressed, but which remain critical for developing an evidence base which can meaningfully inform research, practice, and policy. These insights could help researchers, educators, parents, and policy‐makers to better understand adolescents' digital media use, inform next steps in research, and support the development of balanced policy guidelines that protect adolescents from negative effects, preserve beneficial aspects for their development and well‐being, and respect their emerging autonomy.

## 2 Methods

### 2.1 Participants

The sample consisted of 78 Dutch adolescents aged 12–16 (*M*_age_ = 14.1, *SD*_age_ = 1.1). A total of 10 focus groups were conducted, stratified so that four focus groups included boys (*n* = 34) and six focus groups included girls (*n* = 44). The number of focus groups was determined a priori based on methodological guidelines on saturation in focus group research (Hennink et al. 2019), indicating that the number of focus groups we conducted is sufficient to capture a broad range of experiences and meanings in stratified samples. These adolescents came from two secondary schools in the Netherlands. The educational levels were HAVO and VMBO‐TL; HAVO is a secondary education level that prepares students for higher professional education, while VMBO‐TL is a middle‐level vocational track that prepares students for further vocational education or higher secondary education. The inclusion criterion was being between 12 and 16 years old.

### 2.2 Procedure

The study was approved by the Ethical Review Board of the Faculty of Social and Behavioural Sciences at the University of Amsterdam (FMG‐479). Parental active consent (along adolescents' assent) was provided for adolescents aged 12–15, while adolescents aged 16 provided their own active consent. Information and recruitment letters were sent to schools in the Netherlands, and schools were recruited on a voluntary basis. The teaching coordinators at the schools invited students from different classes that were available to voluntarily participate in the focus groups, resulting in a convenience sample. The focus groups were single sessions consisting of 6–10 adolescents and took place in the participating schools, lasting between 45 and 60 min. Focus groups were chosen as the data collection method because group interactions are optimal to reveal shared subjective norms and meaning‐making around digital media use, which are aspects of adolescents' experiences that remain less accessible through individual or survey‐based methods.

The interviewers were two undergraduate student research assistants who, as part of their internships, learned about the topic of the study, received training in focus group facilitation from the main authors, practised in mock focus groups, received a structured facilitation guide containing prompts and guidance on probing for relevant novel topics, and were supported by a co‐facilitating author in the initial sessions. After each of the focus group interview rounds, the research team had debriefing meetings to evaluate the quality of the process. The interviewers facilitated the discussions with the groups, while another assistant dealt with the audio‐recordings and practical aspects. At the start of the sessions, the interviewers started with an icebreaker and introductions of the group, followed by the discussion of the main topics based on the interview guide. The interview guide was drafted by the authors and contained questions that could be used to probe the three main topics of interest, as well as ideas for boosting engagement and encouraging participants to share perspectives and engage in discussion. The sessions were audio‐recorded using institutional analogue recording devices. Upon completion of the sessions, all participants received a €10 webstore coupon as compensation.

### 2.3 Analysis Plan

The discussions were manually transcribed by the interviewers and then blind‐coded by two coders using ATLAS.ti (version 25.0.1; ATLAS.ti Scientific Software Development GmbH 2025). The coding process combined both inductive and deductive approaches. Codebook thematic analysis (codebook TA) was chosen because the study aimed to examine specific predefined topical domains, while remaining open to how participants articulated experiences within those domains. The first coding cycle was carried out by two graduate research assistants and the first author with a deductive approach, starting with a codebook with predefined topical domains and coding categories based on clinical expertise and theoretical considerations (see Table 1). During this coding cycle, the two coders independently coded the first focus group discussion, after which the research team reflected on the process, discussing how the predefined categories fit to the data, whether any participants' accounts were falling outside of the existing codebook framework, and which new codes warranted addition to the codebook. These iterative rounds of reflection served to evaluate whether the predefined codebook categories adequately captured the meanings in the data and to adjust them if needed, ensuring that the patterns falling outside of the initial framework would not be overlooked. This was repeated after each focus group was coded, and when all the focus groups were coded, the final version of the codebook was created. The final codebook with code occurrence frequencies and total coded segments counts is available in the Supplementary Materials Table S1. This codebook was then used for the second coding cycle, where all the focus group discussions were re‐coded, thereby reflecting a combination of both deductive and inductive approaches characteristic of codebook TA (Braun and Clarke 2021), which differs from reflexive thematic analysis in its use of a structured coding framework while retaining core qualitative aspects of iterative interpretation and researcher reflexivity.

**Table 1:** Predefined coding framework.

Topical domain	Coding categories
Effects of DMU	Positive effects
	Negative effects
Mechanisms of negative DMU effects	Attention
	Self‐Control
	Reward sensitivity
	Time perception
Need for changing DMU	Need to change
	No need to change

*Note:* DMU = digital media use.

The analysis focused on patterns of meaning across the full sample rather than sex‐based comparisons; sex stratification served only a data collection purpose, intended to create a comfortable setting for open discussion, and was not designed for formal sex‐comparative analysis. To support consistency in the application of the broad topical codes during the initial deductive coding phase, intercoder reliability was assessed and found satisfactory (Krippendorff's c‐α‐binary = 0.846; Marzi et al. 2024). While intercoder reliability is not a standard quality criterion in codebook thematic analysis (Braun and Clarke 2021), we used it pragmatically to check for consistency in application of the predefined codebook rather than as an indicator of analytic quality. After coding was completed, patterns of meaning were identified through interpretation and synthesis of the coded material, resulting in thematic findings that were organised within the predefined topical domains.

#### 2.3.1 Positionality Statement

The research team consisted of university researchers with expertise in adolescent development and digital media use. Our interest in the topic stems from ongoing scientific debates about digital media effects on adolescents and the need to better understand the lived experiences of adolescents in this context. The team had no prior relationships with participating schools or students. While our disciplinary knowledge and theoretical focus informed the interview guide and the codebook, we strived to use open‐ended prompts and avoid leading language during the focus groups. We engaged with researcher subjectivity as an active part of the interpretive process: the research team regularly discussed emerging interpretations, divergent patterns in the data, and analytic decisions throughout coding, treating these reflections as integral to the analytic process rather than as a source of bias to be eliminated. Given that the study was exploratory rather than framework‐testing, the analytic process was oriented towards capturing a range of perspectives rather than confirming or disconfirming evidence – diverging accounts were treated as meaningful and illustrative of the breadth of adolescents' experiences with digital media, rather than issues which needed resolution for analytic purposes.

## 3 Findings

### 3.1 Effects of Digital Media Use

#### 3.1.1 Relational and Self‐Processes

A common theme in discussions of digital media use effects was the positive aspect of accessible communication with peers and family, reflected in keeping in touch and sharing updates, as well as enabling efficient planning. Still, some disadvantages were also present, such as miscommunication, exclusion, or pressures to stay available (*Yes, you have arguments much more easily… A friend of mine is ignoring me now over nothing, and that's really annoying*. female [f]). In addition, adolescents expressed disapproval of peers and siblings who spend excessive time online, as well as of parents who are not always good role models regarding digital media use. Social connectedness overlapped with positive aspects of self‐expression and identity formation, as adolescents favourably discussed opportunities to share interests and creative output via digital media. However, there were again concerns voiced, among girls especially, about appearance standards and pressures (*You see very beautiful girls and you kind of look up to them, and then you think, this is how I should be too*. f). Overall, these accounts suggest that digital media use shapes adolescents' self‐processes (e.g., self‐expression and self‐image), as well as relational processes, with frequent interactions between these processes and demands to balance positive and negative aspects.

#### 3.1.2 Mental and Physical Well‐Being

Another common theme was the impact of digital media use on various aspects of well‐being. Experiences of compulsive use (e.g., mindless scrolling) were coupled with feelings of exhaustion or guilt. On the other hand, some adolescents pointed out goal‐oriented use of digital media to calm down, increase positive affect, deal with boredom, or reduce feelings of isolation (*I do homework alone in my room and it's quiet, so I sometimes put a video on for the sound, and I like that – it makes me feel like I'm not alone*. male [m]). Adolescents also described how digital media use affects their physical well‐being. Physical tension was one of the concerns, as it was pointed out that spending time on digital media may not be an adequate form of rest, and that eye strain, headaches, and stress are common symptoms of overuse. Sleep was a common area where negative effects were pointed out, because they would stay up online much later than intended (*You get very little sleep because of it, at least I do. Uh, yesterday I went to sleep at seven o'clock in the morning, because of TikTok*. m). However, some adolescents pointed out that exercise content on digital media inspires them to be physically active. Overall, these accounts point to various ways in which digital media can be used to manage feelings such as boredom or loneliness in the short term, but also how it can lead to negative emotional and physical consequences in the long term when use becomes excessive.

#### 3.1.3 Design Frictions and Functionality

A variety of aspects relating to features of digital media were discussed. Adolescents expressed appreciation of convenience features of digital media for coordinating plans, sharing schoolwork, and quick information access, but at the same time pointed out that this dependence on digital media use for daily tasks can easily sidetrack them into distractions (*If I start doing homework and then go on my phone, I end up doing just a few minutes of homework, and then I'm basically on my phone the whole time*. f). There was frustration about ads, buffering/loading, and constant notifications interrupting functional use. Furthermore, adolescents pointed out that they dislike ‘addictive’ design features (*But every app just has something addictive about it, and that's annoying*. f). Overall, these accounts highlight how features which make digital media useful and efficient can also lead to blurred boundaries between different activities and make adolescents easily lose focus or get stuck with prolonged unintentional engagement with their devices.

#### 3.1.4 Digital Safety and Misinformation

Beyond functional frictions, adolescents also raised significant concerns about safety online. Unwanted contact, inappropriate messages, exposure to cybercrime (i.e., hacking and scams), and privacy concerns were frequently discussed, with girls finding features like blocking helpful. Concerns about loss of control over privately shared content were also prominent (*Everything you send can fall into the wrong hands. It can then be edited, and things can spiral completely out of control*. m). There were also concerns about the accuracy of online information, reflected in misinformation and difficulties determining what information could be trusted online (*Also, when you search on Google you get so many answers, and then you have to go through everything to figure out what the real answer actually is*. f). Overall, these accounts illustrate that adolescents face widespread challenges in the digital world, stemming from features of digital media that expose them to risks, the harmful behaviour of others online, and difficulties determining what information can be trusted.

### 3.2 Mechanisms of Negative Digital Media Use Effects

In discussing the cognitive processes potentially underlying negative digital media use effects, adolescents described a variety of related experiences which made disengagement from digital media use difficult. These experiences were related to attentional capture, difficulties with self‐control, the appeal of rewarding and salient content, and altering perceptions of time during digital media use. These processes often co‐occurred in daily use, rather than being described as isolated instances.

#### 3.2.1 Attentional Capture and Distraction

Adolescents often reported that digital media captured their attention and led to significant time loss, with many describing evenings where time seemed to fly by. Gaming sessions could also last far longer than planned (*I think I'll play for an hour, and then I play a few matches, and I've already been playing for three hours*. m), and participants recognised that digital media are designed to keep them engaged. Multitasking was common and sometimes described as compatible with schoolwork and daily routines (e.g., background music), but videos and social media were seen as too distracting. Some participants said they turned off notifications or put their phones away to focus, but others described risky behaviours such as using their phones while biking (*I know it's not allowed because it's kind of dangerous, but I still do it anyway*. m). Overall, these accounts suggest that digital media frequently captures adolescents' attention in ways that can make sustained focus and disengagement from digital media difficult, especially when the content is visually engaging, socially relevant, or continually available.

#### 3.2.2 Difficulties With Self‐Control

Adolescents described mixed success in controlling their digital media use. They described sometimes being able to stop themselves voluntarily, mostly when there is some other activity that they want to engage in (*if I have to go somewhere, yes, then I stop. Or if I have to eat or something like that, then I will stop*. f). However, they often reported that they lose track of time and exceed their own planned limits on digital media use. Adolescents mentioned setting boundaries, such as no‐phone periods or limits on digital media use at school, but admitted that they often ignore these (*I don't even plan anymore because I know I'll go over it; once I start, I can't stop that easily anymore*. m). Experiences resembling relapse were common, where they described that cravings or habits frequently draw them back into digital media use, even when they want to stop using. These findings indicate that, while some adolescents may feel able to control their digital media use in a way which feels natural, many of them experience difficulties with self‐control that they are aware of, both when it comes to use in the moment and prospective upcoming use.

#### 3.2.3 Appeal of Immediate Rewards and Social Connection

Adolescents highlighted the rewarding nature of digital media use, pointing out that it can quickly improve mood and provide satisfaction through progress or achievements on different platforms (*I was really only doing it for the Snap Score anyway, not because I actually enjoyed sending Snaps or anything*. f). Simultaneously, they acknowledged costs that prolonged use of stimulating digital media content can have, leaving them feeling empty or depleted (*Back when I really used to be on TikTok for like three to four hours a day, afterwards I was really kind of brain‐dead or something. Like all my happiness had just been sucked out of me. (group laughter) Yeah, really!* m). Algorithmic personalisation and endless scrolling seem to keep adolescents engaged by continuously offering novel content, which was described as stimulating and addictive (*You think, I'll just watch two videos. And then you're 20 videos further, and remember ‘oh, I wanted to stop’. And then you keep watching because you find it interesting anyway – what is going to come next?* m), and some noted that this can also reinforce negative moods. Fear of missing out and notifications were described as major drivers for staying online, with many participants expressing a strong desire to remain socially included and keep up to date with their social circle. Overall, adolescents' perspectives suggest that digital media use fosters prolonged engagement through provision of immediate rewards, as well as access to relevant social contexts, but also that this can lead to negative consequences of overuse.

#### 3.2.4 Losing Track of Time

Adolescents reported spending long parts of their days on digital media, particularly after school and before bed, with some describing it as their default activity in free time (*Always after school, just straight away: dropping my bag down on the couch, an hour and a half later dinner with everyone, and then back on my phone*. f). They explained that the short, continuous nature of online content easily prolonged sessions beyond what they had planned (*Then you think, just another half hour, and then it's already an hour and a half later*. f). While many were aware of this overuse, they admitted that digital self‐monitoring tools were not always effective, often leading them to exceed their limits, and in some cases even motivating them to use up their remaining time when they considered stopping early (…*when you see you still have time left, you think ‘I can still go on’, so you just use it up*. m). These findings highlight how time monitoring can be a challenge for adolescents when faced with immersive digital media use, making stopping difficult even despite the intentions to do so.

### 3.3 Need for Change in Digital Media Use

Adolescents' discussions of the need to change their digital media use revealed a contrast between individual motivations to reduce their use and the social and structural contexts that shape (and necessitate) their digital media engagement. Their accounts point towards both individual intentions and perceptions of broader societal expectations as key in determining whether change felt possible and sustainable.

#### 3.3.1 Obstacles to Change and Social Pressures

Adolescents said that reducing digital media use felt like a challenge because of the need to stay socially connected and fears of exclusion, as well as due to practical demands related to schoolwork, communication, and coordination of daily activities. Being offline was described as socially undesirable, leading to missed jokes and participation in group chats, or even bullying (*If you don't have digital media, you are more likely to be bullied, because then you're a bit different; but it's still your own choice to make*. f). Peer norms and trends exerted strong pressure to keep up with what others do, and some clearly indicated that they joined in digital media use mainly to fit in (*If they do it, I do it too; but honestly, it doesn't matter that much to me*. m). Attempts by parents or teachers to impose limits on digital media use were often perceived as frustrating or hypocritical (*Sometimes my dad tells me to get off my phone because I've been on it too long, while he's been on his phone the whole time as well. Then he says we should all put our phones away, but when I look again, he's already back on his phone*. f).

#### 3.3.2 Motivations to Change and Self‐Regulation Attempts

Despite obstacles to change, adolescents expressed clear motivations for change in their digital media use, and these were most often related to getting enough sleep and avoiding fatigue (*I would actually like to reduce it, so that I take more time to sleep instead of being the whole time on my phone or laptop watching YouTube and TikTok*. m). Some described waking up feeling irritable or exhausted after late‐night use, while a few reported being able to voluntarily stop earlier when they had school the next day. Others expressed regret about how much time was lost on digital media use and wished they would spend it differently, on sports, hobbies, or other activities (*You can do much better things than sitting on your phone*. f). Strategies for achieving change included removing apps, leaving phones outside the bedroom overnight, or making collective agreements with friends to take breaks, although many said these were hard to maintain long‐term unless others joined the effort.

#### 3.3.3 Supportive Structures and Constructive Control

Adolescents emphasised that external rules could be both frustrating and helpful. They appreciated when parents encouraged offline activities in a collaborative way, rather than as a form of punishment. Some adolescents even considered it desirable to have shared restrictions that would apply to everyone, saying this would make it easier to step back (*If no one did it anymore, I would be fine with that; but not to suddenly stop on your own*. f). School rules were described as inconsistently enforced, which made them easy to ignore (*Here at school it is actually not allowed, but everyone does it anyway*. f). They pointed out that more consistent expectations from peers, parents, and teachers about their digital media use would make it easier to cut back without feeling isolated.

## 4 Discussion

Despite describing significant self‐regulation difficulties and awareness of the negative consequences of digital media overuse, adolescents in this study consistently framed change as complex and contingent on social and structural conditions beyond individual control. Across 10 focus groups, adolescents experienced digital media use in their daily life as both indispensable and frustrating: it was seen as a relevant source of social connection, self‐expression, and dealing with negative emotions, but also as a source of social pressures, safety concerns, distraction, and compulsive use patterns. The findings showed that positive and negative aspects often occur in proximity, showing that digital media use is deeply embedded in adolescents' everyday lives, which makes the questions of self‐regulation and change more complex. Adolescents appeared motivated to reduce perceived harms of digital media overuse, but their perspectives highlighted that achieving balance is not straightforward, given the social and structural contexts in which digital media use takes place, as well as the developing cognitive processes that shape adolescents' capacity to regulate their digital media use at their age.

Adolescents' emphasis on the utility of digital media for maintaining and fostering social connection aligns with the increased importance and complexity of peer relations during adolescence (Brown and Larson 2009; Nagata et al. 2025). Similarly, the views on self‐expression through digital media align with adolescence being a key period for identity development (Branje et al. 2021; Nagata et al. 2025). Importantly, adolescents' accounts of their lived experiences illustrated how developmental processes like identity development and social development closely intersect in digital media use, as opportunities for self‐expression often occur within peer contexts and related peer/social norms.

Simultaneously, disadvantages within these same domains were discussed as well – communication was sometimes experienced as pressuring or impersonal, and self‐image could be negatively impacted by unrealistic standards and comparisons. Such contrasts within different domains are consistent with previous research showing that digital media use often comes with tradeoffs (Stuart et al. 2022; Valkenburg and Peter 2010; Weinstein 2018). Overall, framing digital media use as a context in which developmental processes take place may explain why adolescents significantly invest in it, despite challenges related to self‐expression, social connection, and well‐being. At the same time, this illustrates how approaches which restrict access without providing alternative contexts for these processes may be experienced as developmentally costly rather than protective.

Along with functions in developmental processes such as peer connection and identity formation, adolescents frequently described struggles with self‐regulation when it comes to digital media use. This aligns with adolescence being a highly dynamic and sensitive developmental period during which emotional, motivational, and social systems rapidly change and impose novel demands, while self‐regulation capacities are still maturing (Dahl et al. 2018). Such a unique developmental position may leave adolescents particularly vulnerable to rewards provided by digital media. Many accounts pointed out that disengagement can be difficult, despite the intentions to stop using digital media. Watching videos or gaming was often described as ways to alleviate boredom or relax, but adolescents commonly reported defaulting to digital media use whenever there was nothing else to do, pointing to a complex interplay between intentional and habitual use. There were accounts of frequent instances of use that went on longer than planned or displaced other activity options. Patterns like this indicate that digital media use occurs on a continuum between purposeful goal‐directed use and more reflexive habitual use, particularly in moments of negative affect (e.g., boredom and loneliness), aligning with behavioural addiction models (Brand et al. 2019) and suggesting challenges with self‐regulation involving inhibitory control and reward‐seeking. Rather than showing a lack of awareness, adolescents seemed to recognise these problematic patterns of digital media use while struggling to prevent them in practice, suggesting that self‐regulation difficulties in this context are not necessarily a motivational problem but a developmental and contextual one.

When reflecting on what makes self‐regulation regarding digital media use a challenge, adolescents discussed processes related to several mechanisms that coalesce to make self‐regulation difficult. Attentional capture coupled with time loss was a common experience, suggesting that highly rewarding digital media content may evoke immersive engagement which interferes with adolescents' ability to monitor and organise time. The rewarding nature of digital content was also emphasised, as a variety of features and affordances, such as novelty, personal relevance, notifications, and gamification, make digital media use more difficult to stop. Experiences of lack of control over both momentary and prospective digital media use were common. These mechanisms were frequently described as co‐occurring, suggesting that cognitive processes such as attentional capture, self‐control, reward sensitivity, and temporal processing, interact to bring about experiences of problematic digital media use. This may suggest theoretical alignment with models of behavioural dysregulation such as the triple‐pathway model (Sonuga‐Barke et al. 2010) and the I‐PACE model (Brand et al. 2019), although the qualitative nature of the data means that such alignment should be interpreted cautiously rather than as evidence in support of these frameworks. At the same time, adolescents' accounts point to intra‐ and inter‐personal processes, such as motivations related to identity and social context, which are not captured by these models and may warrant further theoretical and empirical attention.

In light of these experiences, some adolescents expressed a perceived need for change and discussed a variety of ideas about how to change their digital media use. They were mostly motivated by a desire for better sleep, improved physical well‐being, and opportunities for shared offline activities. Importantly, change was not primarily framed as an individual responsibility. Instead, adolescents stressed the importance of collective and systemic approaches, such as shared agreements with peers, consistent parental and school rules, supportive involvement of others, and even societal change. This emphasis on multi‐level approaches aligns with recent theoretical frameworks arguing that social media interventions, in order to be most effective, need to account for systems beyond the individual level (Skeggs and Orben 2025). Adolescents agreed that reducing digital media use would be more feasible if peers participated in such efforts, suggesting that this could safeguard their concerns about social exclusion and fears of missing out. Structure provided by parents was seen as helpful in regulating digital media use, consistent with research showing that parental rules are associated with less problematic screen use (Nagata et al. 2024), although dissatisfaction was expressed at inconsistencies between parental rules and parental modelling.

These findings highlight the importance of accounting for adolescents' developmental needs when it comes to supporting change. This emphasis aligns with theoretical frameworks on social media interventions grounded in self‐determination theory (Skeggs and Orben 2025). Relatedness and autonomy are especially important in adolescents' accounts, as adolescents actively use digital media to meet those needs, even when such use simultaneously introduces challenges or undermines them (West et al. 2021, 2023). In the context of need for change specifically, the need for relatedness was reflected in adolescents' desire for maintaining social connection with peers, while the need for autonomy was reflected in their preference for collaborative and constructive approaches to change rather than unilaterally imposed restrictions.

When it came to individual‐level solutions, there were mixed views among adolescents in our study: digital monitoring tools were seen as easily bypassed and practically of little utility for regulating use, while more physical strategies (leaving the phone away and deleting apps) were discussed somewhat more favourably. Altogether, these accounts suggest that adolescents in this sample are not without motivation to change their digital media use, but that there is a misalignment between individual self‐regulation efforts and the environment and contexts in which digital media use is embedded as a practical and functional necessity. Importantly, adolescents' emphasis on collective forms of regulation suggests that structural changes should be nuanced and safeguard connectedness and self‐expression, in line with recent evidence which suggests that stricter smartphone bans at schools may reduce connectedness without additionally improving well‐being (Vanluydt et al. 2026).

## 5 Limitations and Strengths

The findings of this study are context‐specific, meaning that the participants were exposed to Dutch cultural norms and school systems, making it possible that views on digital media use could differ in other cultural contexts. Given the convenience sampling method, this sample may also be unrepresentative of adolescents with severe school problems or restrictive home environments (due to requirement of parental consent), that may have prevented them from potential participation in the study. The focus group format is valuable for covering a breadth of experiences, but it may limit deeper insights into private experiences, which may be uncomfortable to share in presence of peers. Finally, adolescents' accounts are time‐contingent and may represent platform‐specific features or current trends, which are subject to quick changes as digital media and societal norms evolve over time.

Despite these limitations, the study makes an important contribution by providing an adolescent‐informed perspective for both research and policy. Adolescent participants were involved as a source of subjective knowledge and reflective about their own behaviour, which places them as main stakeholders in the centre of the research process and ultimately decision‐making processes with regards to digital media use regulations. Focus group methodology allowed for uncovering subjective meaning‐making and shared experiences which remain less accessible with other methodological approaches. The sample representativeness and relevance can also be considered as strengths. Most research samples consist of students in the highest educational levels, while this study involved adolescents from middle and vocational secondary school tracks. Given that adolescence is a period of major developmental change accompanied by an increased risk for the onset of psychopathology (Larsen and Luna 2018; McLaughlin et al. 2015), accounting for adolescents' subjective experiences at this age is highly relevant. The focus on effects, theory‐informed mechanisms, and perspectives on change, provides a comprehensive framework for understanding adolescents' experiences of digital media use in complex developmental and environmental contexts.

## 6 Future Directions

Future research should examine how collective or systemic interventions can best support adolescents in achieving the balance of digital media use that they desire. This could include investigating peer‐based agreements, school‐level policies or consistency of enforcement, and platform design changes that reduce algorithmic pressure and limit persuasive design features such as achievement scores or endless scrolling. Longitudinal and cross‐cultural qualitative studies would be valuable for understanding whether the observed patterns of meanings occur in different educational tracks, cultural contexts, and even age groups. Finally, mixed‐method studies could explore how emotion regulation skills, reward sensitivity, and self‐regulation capacities interact over time to predict digital media overuse, offering informed action points for prevention and intervention efforts when it comes to digital well‐being. This kind of work should aim to explicitly examine how to best address the observed mismatch between adolescents' desires for effective self‐regulation on the one hand, and social, structural, and design features, which are seen as creating vulnerabilities to problematic aspects of digital media use.

## 7 Conclusion

This study highlights that digital media is now one of the central facilitators of developmentally relevant processes, such as identity and social development, alongside other practical and functional features that it provides in navigating daily life. In line with previous research, adolescents experience both positive and negative effects of digital media use. Adolescents highlight a tension between balancing solutions for negative effects with loss of access to developmentally relevant needs, such as connection with peers. They express a desire to change their digital media use, but this seems in conflict with broader social and structural features which make digital media essential to how daily life operates. Therefore, adolescents shared ideas about structural and social changes that could bring about collective change in digital media use, thereby maintaining a context in which social connection remains accessible and exclusion is reduced.

These findings may have implications for both research and policy. They suggest that adolescents' overuse or problematic use of digital media may not only result from insufficient self‐regulation, but also from increased needs that digital media can meet in relation to their developmentally relevant goals. It also indicates that regulations addressing young people's digital media use should consider approaches that provide, support, and ideally improve alternative contexts for engaging in developmental processes. Ultimately, these findings demonstrate that adolescents have a nuanced understanding of the complexity of digital media use and a genuine desire to have balance, which underscores the relevance of their lived experiences not just as a relevant context for research but a necessary input for designing effective solutions. At the same time, they illustrate adolescents' struggles to achieve balance, given the current functional structures in which digital media is embedded as central to everyday activities and functioning.

## Author Contributions

**Luka Todorovic:** conceptualisation, investigation, writing – original draft, methodology, validation, writing – review and editing, software, formal analysis, data curation, project administration, resources. **Annabel Bogaerts:** conceptualisation, investigation, methodology, validation, writing – review and editing, supervision. **Helle Larsen:** conceptualisation, investigation, methodology, validation, writing – review and editing, supervision, resources, project administration.

## Funding

The authors have nothing to report.

## Ethics Statement

The study was approved by the Ethical Review Board of the Faculty of Social and Behavioural Sciences at the University of Amsterdam (FMG‐479).

## Consent

Parental active consent was provided for adolescents aged 12–15, while adolescents aged 16 provided their own active consent.

## Conflicts of Interest

The authors declare no conflicts of interest.

## Supporting information

Supporting File 1

## Data Availability

The data that support the findings of this study are available from the corresponding author upon reasonable request.
