# Supplementary material for: Adolescents' Perspectives on Self‐Regulation and Need for Change in Digital Media Use: A Focus Group Study
Source: J Adolesc. 2026 Jul 6;98(7):2348–58. doi: 10.1002/jad.70220 (PMC13643621; doi:10.1002/jad.70220)
Supplement: Supplementary file 1 [file JAD-98-2348-s001.docx]

**Supplementary Material**

**Table S1**

*Final Codebook*

| Topic | Theme | Code | Occurrence (out of 10) | Coded segments |
| --- | --- | --- | --- | --- |
| effects of DMU | positive effects | communication | 10 | 166 |
|  |  | entertainment | 10 | 178 |
|  |  | information access | 10 | 160 |
|  |  | convenience | 10 | 167 |
|  |  | dealing with boredom | 10 | 103 |
|  |  | self-expression | 8 | 57 |
|  |  | cyber-safety features | 6 | 27 |
|  |  | relaxation | 4 | 31 |
|  |  | health advice | 2 | 8 |
|  | negative effects | communication | 10 | 115 |
|  |  | information access | 9 | 48 |
|  |  | convenience | 10 | 43 |
|  |  | ads | 6 | 53 |
|  |  | technical difficulties | 9 | 110 |
|  |  | cyber-safety | 10 | 377 |
|  |  | addiction | 9 | 127 |
|  |  | health impact | 7 | 49 |
|  |  | self-image | 7 | 63 |
|  |  | psychological impact | 8 | 114 |
|  |  | nuisance | 9 | 31 |
|  | neutral effects | practical dependence | 9 | 118 |
|  |  | non-DMU effects | 9 | 64 |
|  |  | others’ use | 10 | 160 |
|  |  | sleep impact | 10 | 193 |
| mechanisms of negative effects | attention | distraction | 10 | 549 |
|  |  | multi-tasking | 10 | 205 |
|  | self-control | restrained DMU | 10 | 127 |
|  |  | unrestrained DMU | 10 | 260 |
|  |  | self-regulated use | 10 | 115 |
|  |  | unregulated use | 9 | 110 |
|  |  | craving | 10 | 74 |
|  |  | alternatives to DMU | 10 | 228 |
|  | reward | DMU is rewarding | 10 | 98 |
|  |  | DMU is not rewarding | 10 | 77 |
|  |  | fear of missing out | 9 | 61 |
|  |  | novelty | 10 | 62 |
|  |  | achievements | 10 | 47 |
|  |  | personalised content | 7 | 72 |
|  |  | notifications | 9 | 122 |
|  | time perception | time spent | 10 | 460 |
|  |  | monitoring time | 10 | 72 |
|  |  | perceptions of time | 10 | 221 |
|  |  | content duration | 10 | 46 |
| need for changing DMU | need to change | desire to change | 10 | 93 |
|  |  | help to change | 9 | 196 |
|  |  | parental influence | 10 | 166 |
|  |  | sleep as motivator | 6 | 15 |
|  |  | authority control | 10 | 230 |
|  | no need to change | no desire | 10 | 92 |
|  |  | fitting in with others | 8 | 32 |

*Note.* DMU = digital media use. Occurrence = number of focus groups (out of 10) in which the code was applied at least once. Coded segments = total number of coded segments across both coding cycles (counts reflect coding frequency and do not represent unique participant endorsements).
